# Cartilage intermediate layer protein 1 (CILP1): A novel mediator of cardiac extracellular matrix remodelling

**DOI:** 10.1038/s41598-017-16201-y

**Published:** 2017-11-22

**Authors:** Frans A. van Nieuwenhoven, Chantal Munts, Roel C. op’t Veld, Arantxa González, Javier Díez, Stephane Heymans, Blanche Schroen, Marc van Bilsen

**Affiliations:** 1 Department of Physiology, Cardiovascular Research Institute Maastricht (CARIM), Maastricht University, Maastricht, The Netherlands; 2 Program of Cardiovascular Diseases, CIMA, University of Navarra, Pamplona, Spain; 3 CIBERCV, Carlos III National Institute of Health, Madrid, Spain; 4 Department of Cardiology, Cardiovascular Research Institute Maastricht (CARIM), Maastricht University, Maastricht, The Netherlands

## Abstract

Heart failure is accompanied by extracellular matrix (ECM) remodelling, often leading to cardiac fibrosis. In the present study we explored the significance of cartilage intermediate layer protein 1 (CILP1) as a novel mediator of cardiac ECM remodelling. Whole genome transcriptional analysis of human cardiac tissue samples revealed a strong association of CILP1 with many structural (e.g. COL1A2 r^2^ = 0.83) and non-structural (e.g. TGFB3 r^2^ = 0.75) ECM proteins. Gene enrichment analysis further underscored the involvement of CILP1 in human cardiac ECM remodelling and TGFβ signalling. Myocardial CILP1 protein levels were significantly elevated in human infarct tissue and in aortic valve stenosis patients. CILP1 mRNA levels markedly increased in mouse heart after myocardial infarction, transverse aortic constriction, and angiotensin II treatment. Cardiac fibroblasts were found to be the primary source of cardiac CILP1 expression. Recombinant CILP1 inhibited TGFβ-induced αSMA gene and protein expression in cardiac fibroblasts. In addition, CILP1 overexpression in HEK293 cells strongly (5-fold p < 0.05) inhibited TGFβ signalling activity. In conclusion, our study identifies CILP1 as a new cardiac matricellular protein interfering with pro-fibrotic TGFβ signalling, and as a novel sensitive marker for cardiac fibrosis.

## Introduction

In cardiac tissue, cardiomyocytes, fibroblasts, vascular and immune cells are embedded in the myocardial extracellular matrix (ECM), which provides structure, transmits mechanical forces and modulates cell function^1^. The myocardial ECM is mainly composed of fibrillar collagen and the balance of ECM synthesis and degradation is governed by cardiac fibroblasts. Accumulation of ECM, finally resulting in cardiac fibrosis, is a common feature of many forms of heart disease^1,2^. The cardiac ECM not only consists of structural proteins like the collagens, but also of various non-structural proteins that modulate ECM properties, including connective tissue growth factor (CTGF, CCN2) and Osteonectin^1,3,4^. Many of these so-called matricellular proteins were first described to have a function in bone formation and homeostasis and later found to be involved in cardiac disease progression^4^. Transcriptome analyses of mouse hearts after transverse aortic constriction revealed increased expression of cartilage intermediate layer protein 1 (CILP1)^5^. This protein has not been linked to cardiac pathophysiology previously and is only known for its role in cartilage^6,7^.

The CILP1 gene codes for a precursor protein which is secreted and cleaved into a N-terminal and a C-terminal part^7–10^. The N-terminal fragment corresponds to the CILP1 protein, while the C-terminal portion is homologous to nucleotide pyrophosphohydrolase (NTPPHase)^9,10^. CILP1 is an extracellular matrix (ECM) protein abundant in articular cartilage^6^ and has been implicated in several diseases affecting the cartilage^11–13^. In chondrocytes, TGFβ induces CILP1 expression^14^ and CILP1 was shown to inhibit TGFβ-mediated induction of ECM genes in nucleus pulposus cells^13^. Previously, CILP1 has been shown to be “involved” in cartilage degenerative diseases^11^. More recently, CILP1 was detected in human skeletal and cardiac muscle tissue^15,16^. Using proteomics, Barallobre-Barreiro and colleagues identified CILP1 as one of many proteins present in porcine ECM following ischemia/reperfusion injury^15^.

The aim of the present study was to investigate the effect of cardiac disease on CILP1 expression and to determine the role of CILP1 in myocardial remodelling in both man and mice. Whole genome transcriptional analyses on human myocardial samples from patients with aortic valve stenosis were performed to determine whether CILP1 expression was associated with cardiac fibrosis. Myocardial CILP1 expression was assessed in mouse models of myocardial infarction and hypertension. The cellular source of myocardial CILP1 expression was shown to be cardiac fibroblasts and the regulation of CILP1 expression was studied in isolated human and rat cardiac fibroblasts. Finally, the effect of CILP1 on TGFβ signalling was examined *in vitro*.

## Results

### CILP1 is expressed in human heart and correlates strongly with extracellular matrix proteins

**Figure 1:**
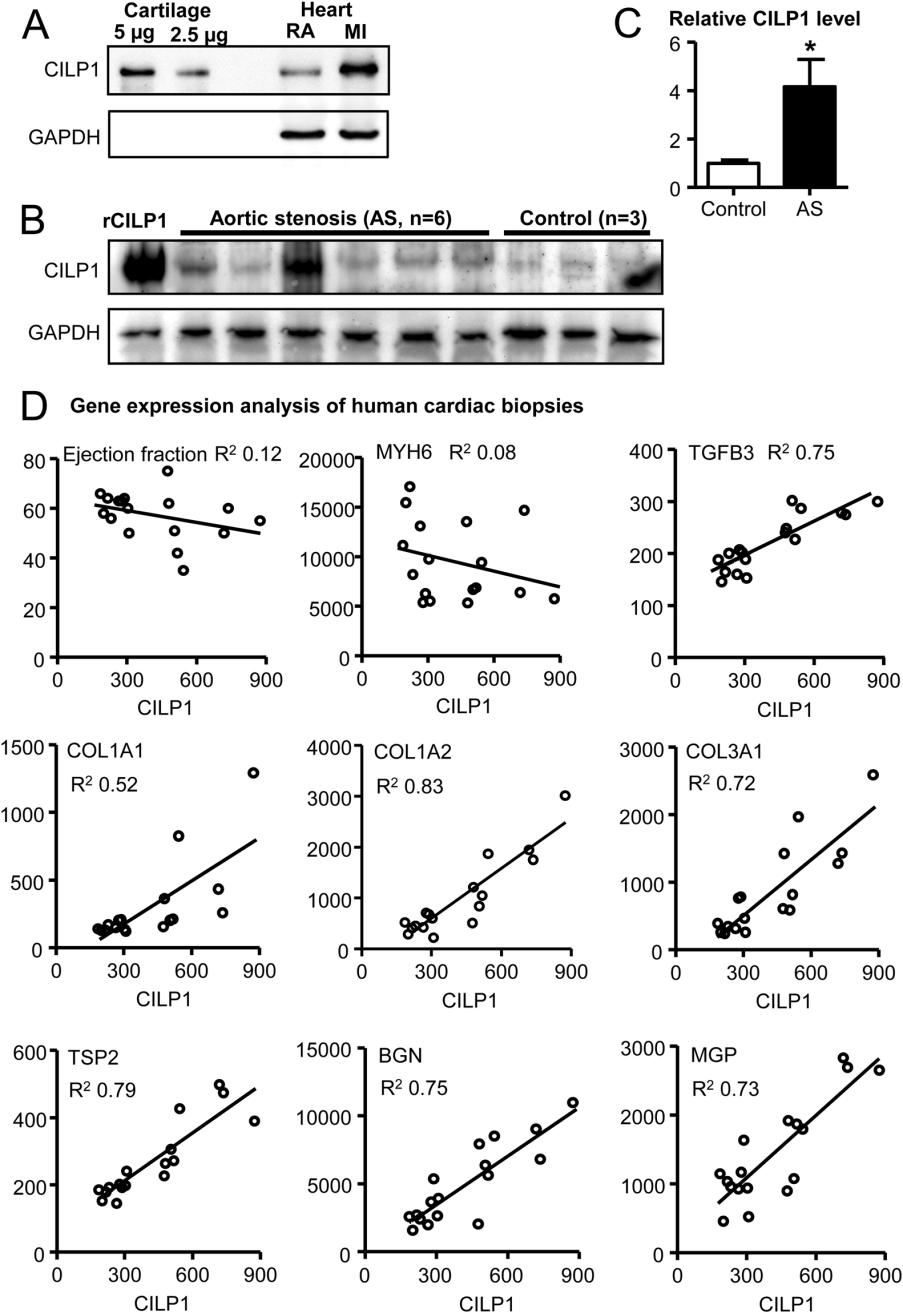
CILP1 expression in human cardiac tissue. (**A**) Increased CILP expression in human myocardial infarct tissue. CILP1 protein was detected in human myocardial tissue by Western blot analysis. RA; remote area tissue, MI; myocardial infarct tissue. Note that the tissue samples from RA and MI were obtained from the same human subject. Cartilage was included as a positive control for CILP1. (**B**) Western blot of endomyocardial biopsies from patients with aortic valve stenosis and control subjects. Lane 1, Control subject spiked with 1 ng recombinant CILP1; Lanes 2–7 Aortic valve stenosis (AS) patients; Lanes 8–10, control subjects. (**C**) Quantification of the CILP1 signal from the Western blot shown in B. The signal of CILP1 was corrected for the corresponding GAPDH signal. CILP1 protein levels are shown as mean ± SEM, relative to control subjects. (**D**) Gene expression analysis of cardiac biopsies from human aortic valve stenosis patients (n = 11) and patients undergoing CABG (n = 6). Pearson correlations were calculated between CILP1-expression and ejection fraction (%), and the expression of the following genes: MYH6, TGFB3, COL1A1, COL1A2, COL3A1, TSP2, BGN and MGP. All expression levels are expressed as normalized intensities.

Western blot analysis of human myocardial tissue samples was performed to assess if CILP1 protein is expressed in cardiac tissue. CILP1 protein was clearly present in myocardial tissue samples, at the expected molecular mass of ±90 kDa (Fig. 1A). The CILP1 protein signal was stronger in infarct tissue as compared to remote non-infarcted myocardial tissue from the same patient. In addition, myocardial CILP1 protein levels were higher in patients with aortic valve stenosis as compared to controls (Fig. 1B,C). Microarray analysis was performed on LV tissue samples from control patients and patients with aortic stenosis. Relative CILP1 expression levels tended to be higher in patients with an ejection fraction <55% (supplemental Fig. S1). All genes correlating strongly with CILP1 (r2 > 0.5, n = 281) are shown in supplemental table S3. Subsequent enrichment analysis using this subset showed that these genes were primarily involved in processes associated with cardiac fibrosis, such as collagen catabolic process, extracellular matrix disassembly, extracellular matrix organization (supplemental table S4). In addition, TGFβ signalling genes were overrepresented. Figure 1D shows that CILP1 correlated poorly to ejection fraction (marker for cardiac function) and MYH6 (marker of hypertrophy), but very strongly with TGFB3, structural ECM proteins like collagens COL1A1, COL1A2, COL3A1, and matricellular proteins like thrombospondin 2 (TSP2), biglycan (BGN), and matrix Gla protein (MGP).

### CILP1 expression is high in myocardial remodelling

**Figure 2:**
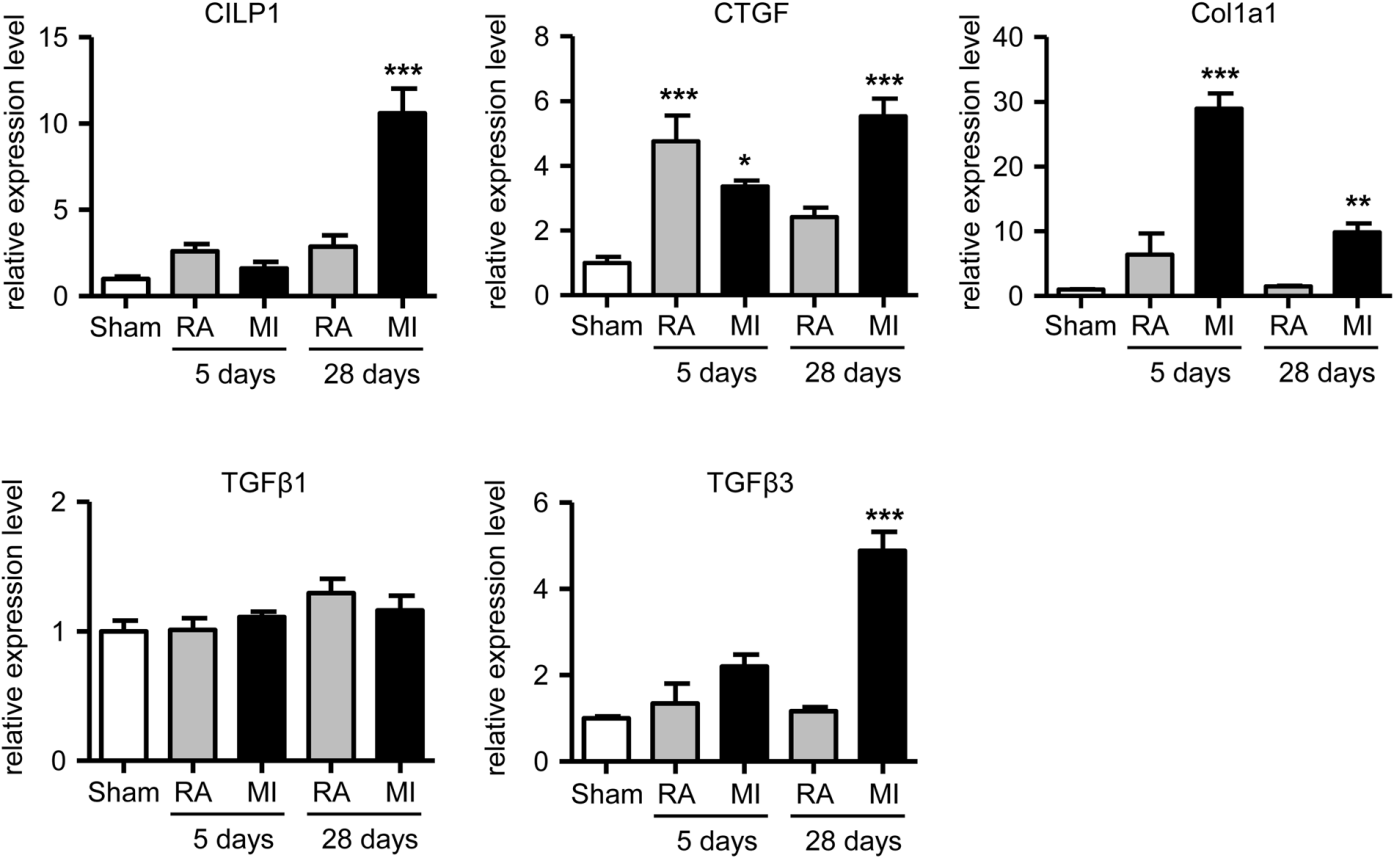
Cardiac left ventricular expression level of CILP1, CTGF, Col1a1, TGFβ1 and TGFβ3 in sham animals (C, n = 6) and in the infarct tissue (MI) and remote area (RA) of animals at 5 days (n = 4) and 28 days (n = 7) after induction of a myocardial infarction (MI). Data were normalized to the housekeeping gene Cyclophilin-A using the comparative threshold cycle (Ct) method by calculating 2^ΔCt^. Data are expressed relative to the sham animals as mean ± SEM. **p < 0.01; ***p < 0.001.

CILP1 expression was determined in mouse LV infarct tissue (MI) and remote non-infarcted area (RA) at 5 days and 4 weeks following myocardial infarction and compared to connective tissue growth factor (CTGF), collagen type I (COL1A1), TGFβ1 and TGFβ3. As shown in Fig. 2, CILP1 expression is strongly increased in infarct tissue at 4 weeks following infarction. The strongest increase in COL1A1 expression was observed at 5 days, while expression levels of TGFβ3 and CTGF showed a gradual increase at 5 day and 4 weeks. Surprisingly, TGFβ1 expression was not affected at the time points studied. In the remote area, CILP1, COL1A1, TGFβ1 and TGFβ3 did not show any significant changes at 5 and 28 days following the myocardial infarction. CTGF expression was increased significantly at 5 days, but not at 28 days (Fig. 2). Myocardial CILP1 expression was also found to be significantly upregulated after both angiotensin-II treatment (supplemental Fig. S2) and transverse aortic constriction (supplemental Fig. S3), indicating that CILP1 is involved in myocardial remodelling induced by increased cardiac afterload.

### Fibroblasts are the main cardiac source of CILP1

**Figure 3:**
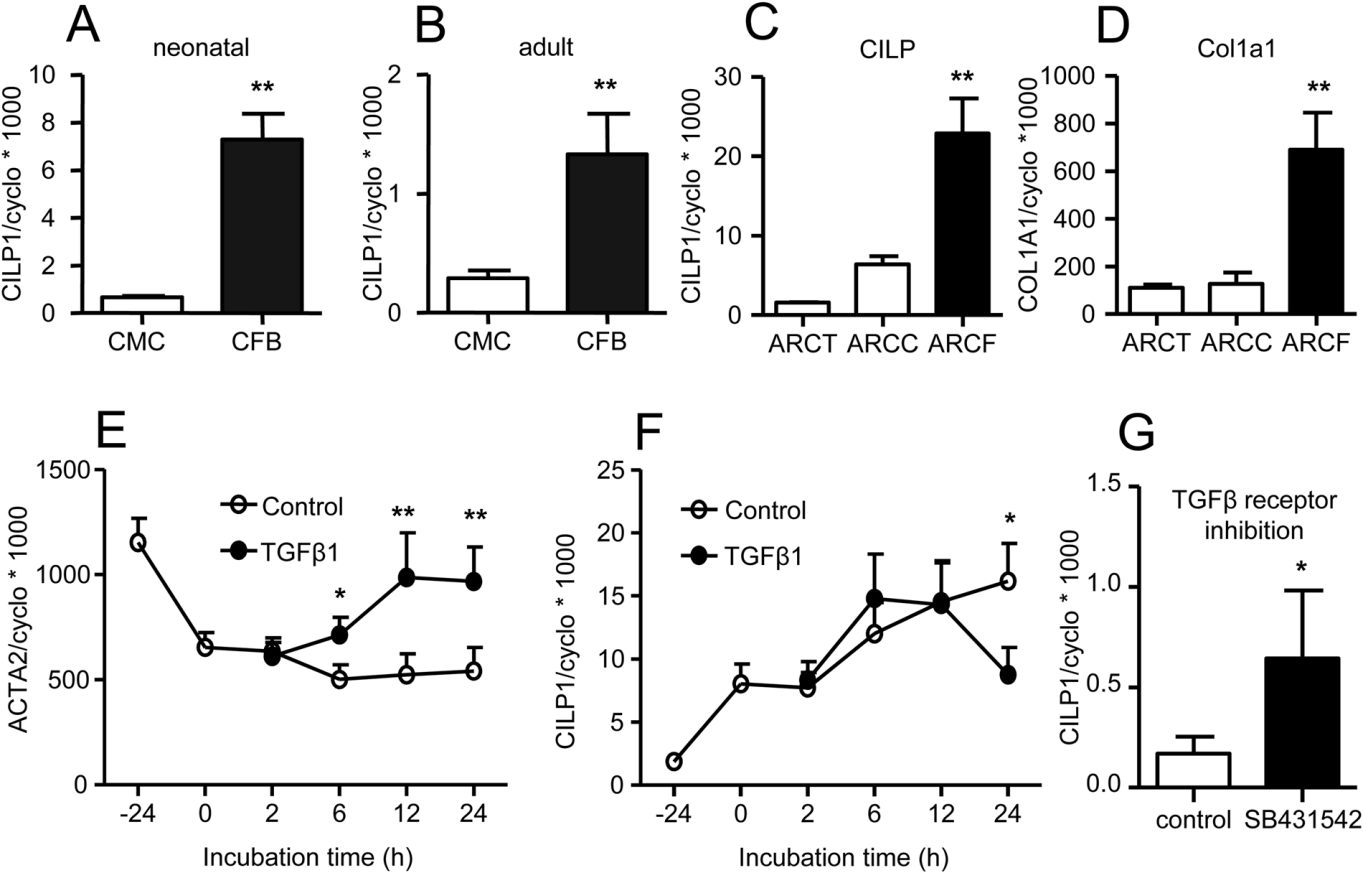
CILP1 expression levels in isolated cardiac cells. **(A)** CILP1 mRNA levels in adult rat cardiomyocytes (CMC) and cardiac fibroblasts (CFB) (n = 8–14 isolations). (**B**) CILP1 mRNA levels in neonatal rat cardiomyocytes (CMC) and cardiac fibroblasts (CFB) (n = 8–14 isolations). **(C)** CILP1 and (**D**) Col1A1 expression during isolation of adult rat cardiac fibroblasts; ARCT: adult rat cardiac tissue (n = 3), ARCC: adult rat cardiac cells (after collagenase, but before adhesion to culture flasks, n = 4), ARCF: adult rat cardiac fibroblasts (immediately after 1 hour attachment to culture flasks, n = 3). (**E** and **F**) Effect of serum-starvation and TGFβ1 on ACTA2 and CILP1expression levels in adult rat cardiac ventricular fibroblasts (n = 5 separate experiments). **(G)** Effect of TGFβ type 1 receptor inhibitor SB431542 for 24 hrs on adult rat cardiac fibroblast CILP1 expression levels in the presence of serum (n = 4). Data were normalized to the housekeeping gene Cyclophilin-A using the comparative threshold cycle (Ct) method by calculating 2^ΔCt^ and multiplied by 1000 (formula 1000 * 2^ΔCt^), to enhance readability. Data are expressed as mean ± SEM. *p < 0.05; **p < 0.01.

To determine the cellular source of CILP1, we analysed its expression in isolated primary rat cardiomyocytes (CMC) and cardiac fibroblasts (CFB). As shown in Fig. 3A,B, CILP1 expression level was much higher in CFB than in CMC, both in neonatal as well as adult cells. To rule out possible effects of culture, we tested CILP1 expression levels in tissue and cells at various stages during the isolation procedure. During the isolation procedure fibroblast-enriched fractions showed strongest CILP1 expression, together with fibroblast marker COL1A1 (Fig. 3C,D), further confirming that fibroblasts were the main source of myocardial CILP1 expression.

### CILP1 expression in primary adult cardiac ventricular fibroblasts (CFB)

Regulation of CILP1 expression was studied in rat primary adult cardiac ventricular fibroblasts (CFB). Importantly, CILP1 expression dropped strongly upon culturing in serum-containing medium, and serum starvation significantly increased CILP1 expression (Fig. 3F). By contrast, the expression of alfa-smooth muscle actin (αSMA, ACTA2) was high in serum-containing culture media, and its expression decreased upon serum starvation (Fig. 3E). TGFβ1 increased αSMA expression, but inhibited CILP1 expression in CFB. Inhibiting TGFβ-receptor type 1, increased CILP1-expression in CFB cultured in serum-containing medium, indicating that CILP1 downregulation by serum was at least partly due to TGFβ (Fig. 3G). CILP1 expression in CFB was also decreased by IGF1, but unaffected by IL-1a (supplemental Fig. S4).

**Figure 4:**
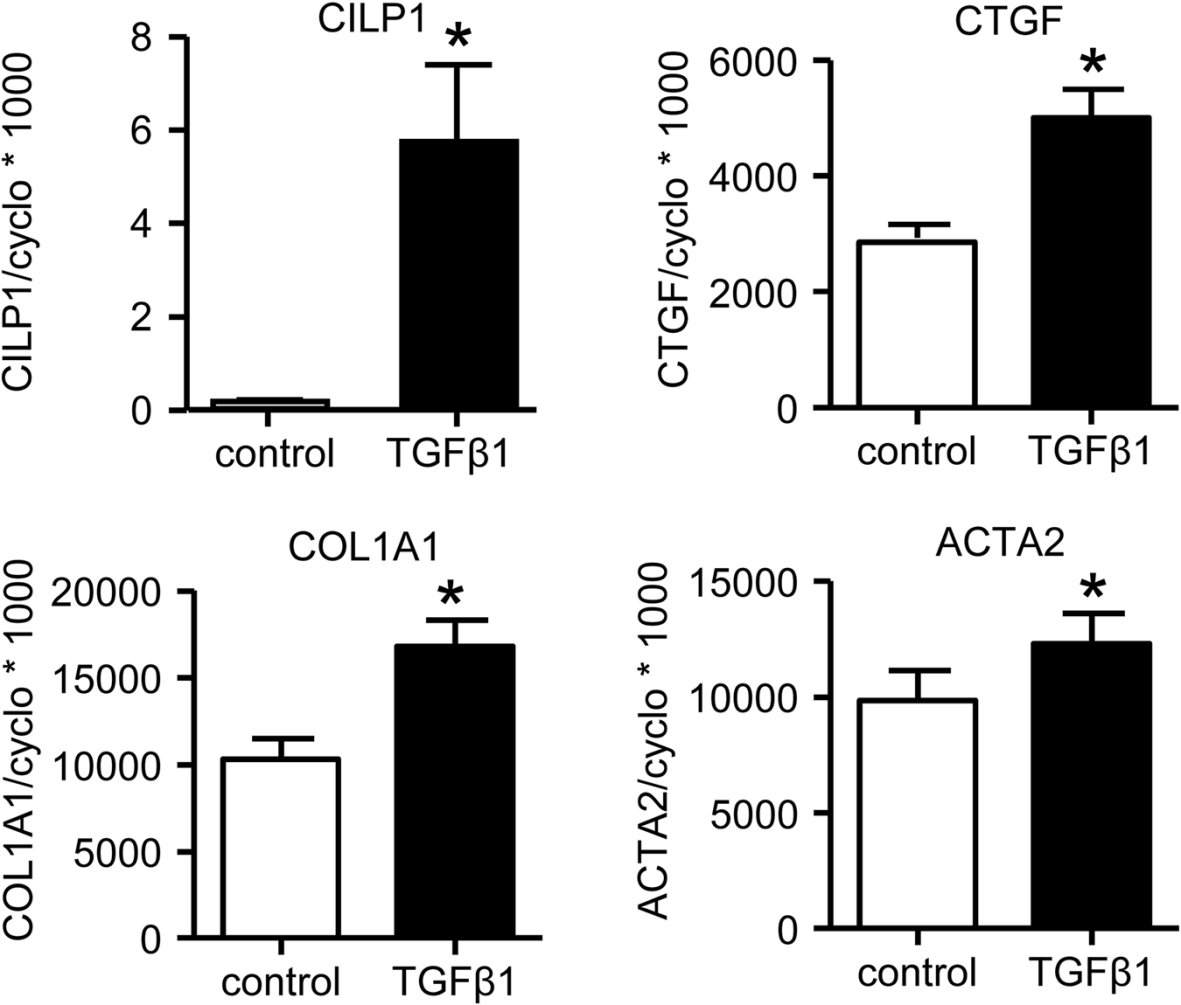
Effect of TGFβ1-incubation for 24 h on gene expression levels of CILP1, CTGF, Col1a1 and TGFβ1 in human cardiac atrial fibroblasts isolated from 3 patients. Data were normalized to the housekeeping gene Cyclophilin-A using the comparative threshold cycle (Ct) method by calculating 2^ΔCt^ and multiplied by 1000 (formula 1000 * 2^ΔCt^), to enhance readability. Data are expressed as mean ± SEM. *p < 0.05.

In stark contrast to the observed TGFβ1-induced CILP1-decrease in rat CFB, we observed a strong increase in CILP1 expression by TGFβ1 in human atrial fibroblasts (Fig. 4). Of note is that these atrial fibroblasts were cultured for several weeks and were strongly differentiated towards myofibroblasts, as can be deduced from the high expression of ACTA2 (αSMA). Baseline CILP1 expression in these atrial fibroblast was very low, most likely caused by prolonged culturing. Still these human atrial fibroblasts showed a clear TGFβ1-induced increase of COL1A1 and CTGF expression (Fig. 4).

### CILP1 inhibits TGFβ activity

**Figure 5:**
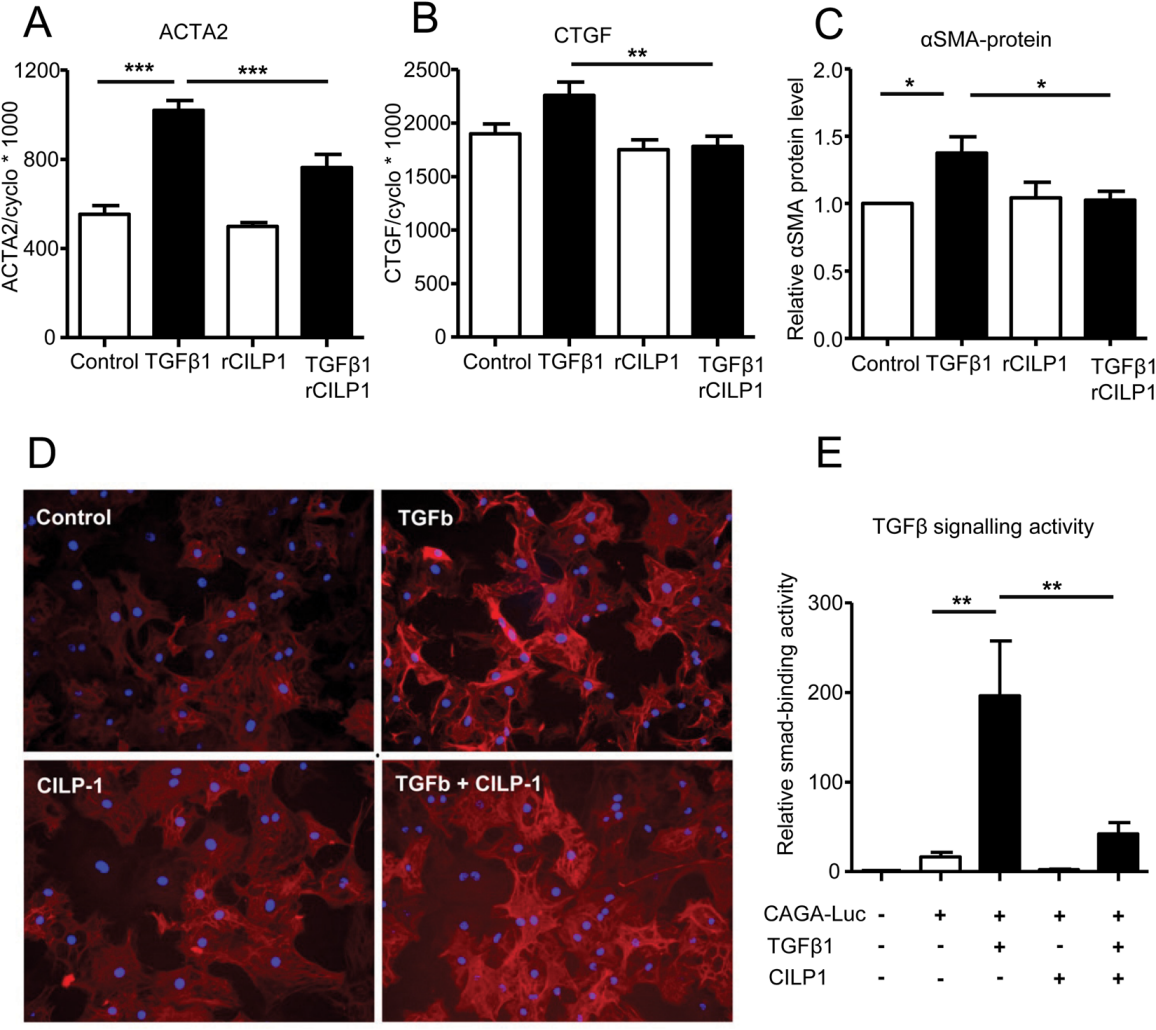
Inhibitory effect of CILP1 on TGFβ1 signalling. (**A**) Inhibitory effect of recombinant CILP1 on TGFβ1-induced ACTA2 overexpression in adult rat CFB (treatment 24 hrs, data from 6 separate cell isolations). (**B**) Inhibitory effect of recombinant CILP1 on TGFβ1-induced CTGF overexpression in adult rat CFB (treatment 24 hrs, data from 6 separate cell isolations). Data from A en B were normalized to the housekeeping gene Cyclophilin-A using the comparative threshold cycle (Ct) method by calculating 2ΔCt and multiplied by 1000 (formula 1000 * 2ΔCt), to enhance readability. (**C**) Relative αSMA protein level as determined by ELISA in adult rat CFB treated for 48 hrs with TGFβ1, CILP1 or the combination (data are shown relative to control from 6 separate cell isolations). (**D**) typical examples of αSMA staining in adult rat CFB incubated for 48 hrs with TGFβ1, CILP1 or the combination. (**E**) Effect of CILP1 on TGFβ-signalling using a TGFβ promoter/reporter assay. HEK293 cells were cotransfected with CILP1 and the TGFβ promoter/reporter construct, containing SMAD-binding elements (CAGA-Luc) and incubated with TGFβ1 for 24 h before analysis of SMAD-binding activity (data are shown relative to control from 5 separate experiments). All data are expressed as mean ± SEM. *p < 0.05, **p < 0.01, ***p < 0.001.

Recombinant CILP1 inhibited the TGFβ-induced increase of both ACTA2 (αSMA) and CTGF expression in CFB (Fig. 5A,B). In addition, staining of αSMA indicated lower intensity in TGFβ-treated cells exposed to CILP1 as compared with TGFβ alone (Fig. 5D). CILP1 completely blocked the TGFβ-induced increase in αSMA-protein level (Fig. 5C). To study if the inhibitory effect of CILP1 on TGFβ action was through SMAD signalling, we co-transfected HEK293 cells with a TGFβ promoter/reporter construct consisting of SMAD binding elements (CAGA-Luc, see ref^17^) and a CILP1 expression vector. Western blot analysis showed that transfection of HEK293 resulted in the abundant secretion the ±90 kDa CILP1 protein into the medium, indicating that the CILP1 protein was expressed, secreted and cleaved from the full-length precursor protein (supplemental Fig. S5). Co-transfection of CILP1 together with a TGFβ-promoter-reporter construct containing SMAD binding elements^17^ showed that SMAD-binding was enhanced by TGFβ1, but strongly inhibited by CILP1 (Fig. 5E). This shows that CILP1 overexpression inhibited TGFβ1 activity via interference with SMAD signalling.

## Discussion

The present study identified cartilage intermediate-layer protein 1 (CILP1) as a novel mediator of cardiac ECM remodelling. Expression of CILP1 is highly associated with that of other ECM genes and with genes involved in TGFβ signalling in human cardiac tissue. Cardiac CILP1 expression was strongly increased in diverse mouse models of cardiac ECM remodelling. In cell experiments, CILP1 inhibited TGFβ activity. Together, our results indicate that CILP1 is a novel matricellular protein involved in ECM remodelling in human heart disease and a sensitive marker for cardiac fibrosis.

We show that cardiac CILP1 expression is strongly elevated in human infarct tissue and in preclinical models of myocardial infarction and pressure overloading of the heart, such as transverse aortic constriction and angiotensin II infusion. Although Barallobre-Barreiro and colleagues previously showed immunofluorescent staining of CILP1 in human cardiac tissue^15^, we are the first to clearly correlate CILP1 expression levels with ECM remodelling in human cardiac samples using microarray and subsequent gene enrichment analysis. CILP1 expression appears to be very sensitive to changes in the cardiac ECM, irrespective of the initial cause, making it an ideal marker for cardiac ECM remodelling.

Cardiac CILP1 expression in the aortic stenosis patients was very strongly correlated to TGFβ3. Also in our infarcted mouse hearts, TGFβ3 was increased after 4 weeks in the infarct region, together with CILP1. By contrast, TGFβ1 expression remained stable in infarcted and remote regions in both time points examined. This is remarkable as previous studies have reported increased TGFβ1 expression, especially during the first 3 days following myocardial infarction (recently reviewed)^18^. Also in the aortic stenosis patients used for our western blot analyses, myocardial TGFβ1 expression was increased^19^. Possibly, we have missed the temporary increase in TGFβ1 expression in our study. Moreover, inactive TGFβ1 already present in the myocardium might become activated by release from the latent complex following infarction^18^, so TGFβ1 activity can be increased in the absence of changes in gene expression. Therefore, TGFβ1 might have played a role in the observed induction of CTGF and Col1a1 at the 5 day time point. Although *in vitro* studies indicate that different isoforms of TGFβ have similar effects^18^, in the physiological context of the body, TGFβ1 and TGFβ3 most likely have unique roles in the tissue response to injury.

Our study clearly shows that fibroblasts are the primary source of CILP1 in the heart. In isolated cardiac fibroblasts CILP1 expression is strongly influenced by culturing conditions. We observed a strong inhibition of CILP1 expression by serum. Moreover, we identified serum factors, such as IGF1 and TGFβ, which play a role in decreasing CILP1 expression in cultured CFB. The observed reduction of CILP1 expression in primary rat ventricular fibroblasts is in contrast to the strong TGFβ-induced increase in CILP1 levels we found in human primary atrial fibroblasts. The latter results are in line with previous research in chondrocytes, where TGFβ was also shown to induce CILP1 expression^20^. The discrepancy between the rat ventricular and human atrial fibroblasts could be caused by the different origin and species, but might also be explained by the strong effect of prolonged culturing on CILP1 levels. CILP1 expression in the human atrial fibroblasts was almost undetectably low under these conditions, and in this condition TGFβ treatment strongly elevated CILP1 levels. Similarly, we tested commercially available human ventricular cardiac fibroblast cells and the mouse fibroblast cell line 3T3, and both showed a very low baseline CILP1 expression and strong induction of CILP1 by TGFβ. In the freshly isolated rat CBF however, CILP1 expression was consistently much higher, and here TGFβ induced a moderate but statistically significant reduction in CILP1 expression. The biological significance of these contradicting *in vitro* results merits further investigation.

We showed that recombinant CILP1 inhibits TGFβ-induced ECM protein expression in adult cardiac fibroblasts. Furthermore, overexpressing CILP1 attenuated TGFβ signalling in HEK293 cells. This is in accordance with previous results observed in nucleus pulposus cells^13^. The latter study also showed direct binding of CILP1 to TGFβ *in vitro* and colocalization of CILP1 and TGFβ in human intervertebral discs *in vivo*, and it is argued that CILP1 regulates various functions of TGFβ^13^. The time frame of induction of CILP1 following myocardial infarction (see Fig. 2) shows that after 5 days CILP1 induction is not significant, in contrast to the strongly increased COL1A1 (almost 30-fold) or CTGF (almost 4-fold). At 4 weeks after myocardial infarction, however, CILP1 levels are very strongly elevated (10-fold) while COL1A1 levels at this time-point are already decreasing. This indicates that CILP1, possibly by inhibiting TGFβ signalling activity, would provide a brake on the ECM accumulation and prevent excessive cardiac fibrosis. Thus, our results indicate that cardiac CILP1 might act as an endogenous modulator of TGFβ-signalling. Given the importance of TGFβ in myocardial ECM remodelling, CILP1 is an attractive target for future research.

This study was designed to show the association of CILP1 with cardiac ECM remodelling in human heart disease. However, since no interventions of CILP1 expression were investigated, a definite causal role for CILP1 in cardiac ECM remodelling remains to be proven. Further, it is important to note that the CILP1 gene codes for a precursor protein which is cleaved into CILP1 protein (N-terminal fragment) and nucleotide pyrophosphohydrolase (NTPPHase, C-terminal fragment). It is not clear at the moment, if increased gene expression and mRNA levels of CILP1 would lead to similarly increased levels of both proteins, and what could be the role of the putative NTPPHase in the heart. Future studies should be designed to specifically target cardiac CILP1 and the NTPPHase-like C-terminal fragment to provide insight into their separate roles in the heart. Moreover, the induction of proteins in the remodelling heart that are highly expressed in cartilage such as CILP-1 and CTGF deserves attention. Possibly, factors like hypoxia or changes in osmolarity that are present both in the remodelling heart and in cartilage might be important in driving the expression of cartilage-related genes in the heart, and studies addressing the regulation of this process are warranted.

In conclusion, the present study shows that CILP1 is expressed in the human heart, correlates strongly with ECM remodelling and TGFβ-signalling, and is elevated in cardiac disease models. Cardiac fibroblasts are the main source of CILP1 and our *in vitro* experiments show that CILP1 inhibits TGFβ-activity. Together, our results indicate that CILP1 is a novel, sensitive marker of cardiac fibrosis, which seems to act as an autocrine inhibitor of this process.

## Methods

### Human and animal tissue collection

All tissue samples from patients were obtained in accordance with the relevant guidelines and regulations, after written informed consent and under approval from the Institutional Ethics Committee of the Virgen de la Victoria University Hospital (Malaga, Spain), or from the Institutional Ethics Committee of the University Hospital of Leuven. All animal experiments were approved by the Institutional Animal Ethics Committee of the Maastricht University and performed according to European Union guidelines (legislation on the protection of animals used for scientific purposes: Directive 2010/63/EU).

### Western blot analysis of human cartilage and cardiac tissue samples

For Western blot analyses of CILP protein, human cartilage was obtained during knee surgery and myocardial infarct tissue was collected post-mortem. Homogenization of human tissue was performed using a micro-dismembrator (Braun Biotech International). 10% tissue homogenates were made in sample buffer (2% SDS, 10% glycerol, 50 mM Tris). In addition, myocardial CILP-1 protein levels were determined in a subgroup of patients (n = 6) from a cohort of patients with severe aortic valve stenosis previously described^19^. Endomyocardial biopsies were obtained at the time of aortic valve replacement. As controls, endomyocardial biopsies were obtained from autopsies of subjects with no macroscopic and microscopic cardiac lesions (n = 3).

Protein extracts were separated on a 10% SDS-PAGE gel under reducing conditions. Proteins were transferred to PVDF membrane (Immobilon 0.45uM; Millipore) for immunodetection. After blocking, the membrane was probed with anti-human CILP-1 N-terminal peptide antibody (R&D Systems). Bound antibodies were detected with HRP conjugated secondary antibody (rabbit-anti-goat-HRP; DAKO) and illuminated by incubating the membrane with Western Blotting Detection Reagent (SuperSignal, Pierce). The CILP1 Western blot was quantified using Image J software and the signal of CILP1 was corrected for the corresponding GAPDH signal. Subsequently, all values were divided by the average of the 3 control samples to obtain the CILP1 protein levels relative to control.

### Microarray analysis of human myocardial tissue samples

Transmural needle biopsies (1–2 mg tissue) from the cardiac anterior left ventricle were obtained as described previously^21^ from 11 patients with aortic valve stenosis (AS). Patients undergoing coronary artery bypass graft (CABG) surgery (n = 6) served as controls. Clinical data of the patients are shown in supplemental table S1. RNA was isolated using the RNeasy kit (Qiagen) and quality of RNA was determined using Bioanalyser Nanochip (Agilent Technologies). RNA purity and concentration was measured with the NanoDrop Spectrophotometer (Nanodrop Technologies). The human total RNA was hybridized to Illumina Human miRNAv2 Expression Panel arrays by ServiceXS (Leiden, The Netherlands). The lumi 2 package from Bioconductor was used to annotate and quantile normalize the expression data. To assess differential expression, the limma 3 package was used and genes with an adjusted p-value of <0.05 were considered significantly differentially regulated. With the genes of which the expression correlated strongly (r2 > 0.5) with CILP1, a statistical overrepresentation test was performed using the PANTHER classification system^22^.

### Mouse models of heart disease

Cardiac CILP1 expression levels were studied in diverse preclinical models of myocardial remodelling. Adult C57Bl6/J mice (n = 17) were subjected to regional myocardial infarction by occlusion of the left anterior descending coronary artery (n = 11) and control animals were sham operated (n = 6) as described previously^23^. RNA was isolated from the infarct area and remote non-infarcted area were obtained at 5 days (n = 4) or 28 days (n = 7) after infarction. In addition, myocardial RNA samples of mice after transverse aortic constriction^24^ and angiotensin II induced hypertension^25^ were used in this study. Transverse aortic constriction or sham surgery was performed in 2-month-old mice by placing a defined 27-gauge constriction between the first and second truncus of the aortic arch. Mice were sacrificed 4 weeks following the intervention^24^. Angiotensin II was administered in mice at 14 week of age via subcutaneously implanted osmotic minipumps (ALZET, CA, USA) at a delivery dose of 1 mg/kg per day, and the mice were sacrificed after 4 weeks^25^.

### Isolation and culture of cardiac cells

Human and rat cardiac fibroblasts were isolated and cultured as described previously^26,27^. In short, cardiac tissue was washed and digested using collagenase (Worthington type II). Fibroblasts were separated from other cardiac cells by adherence for 1 hour to cell culture flasks. Primary fibroblasts were used up to passage 3. To determine regulation and effects of CILP1, cells were serum-starved for 24 hours before incubation with TGFβ1 (1 ng/ml), IL-1a (1 ng/ml), IGF1 (100 ng/ml) and CILP1 (100 ng/ml) for the indicated time (All obtained from R&D Systems). Adult and neonatal cardiomyocytes were isolated and cultured as described previously^28,29^. To inhibit TGFβ type 1 receptor, cells were incubated in serum-containing culture medium for 24 hrs with SB431542 (10 µM, Sigma).

### Gene expression analyses

Total RNA was isolated from cells or tissue samples using RNeasy kits (Qiagen) and reversed transcribed into cDNA using the iScript cDNA synthesis kit (Biorad) according to the manufacturer’s protocol. Real-time PCR was performed on an iCycler accompanied by the My IQ single color real-time PCR detection system using iQ SYBR-Green Supermix (Biorad). Gene expression levels of alpha-Smooth muscle actin (ACTA2), Cartilage intermediate-layer protein 1 (CILP1), Collagen, type 1, alpha 1 (Col1A1), Connective tissue growth factor (CTGF), Transforming growth factor, beta 1 (TGFβ1), Transforming growth factor, beta 3 (TGFβ3) were normalized using the housekeeping gene Cyclophilin-A, and their relative expression was calculated using the comparative threshold cycle (Ct) method by calculating 2^ΔCt^ (e.g. 2^Cyclophilin Ct – CILP1 Ct^). The gene expression levels in the animal experiments were all presented as relative to the control (sham) group, which was set at 1. In the isolated cell culture experiments, gene expression values were multiplied by 1000 (formula 1000 * 2^ΔCt^), to enhance readability. The sequences of the specific primers used are provided in Supplemental Table S2.

### αSMA staining and ELISA

αSMA staining was performed essentially as described previously^27^. Serum-starved primary adult rat fibroblasts were cultured in 8-well LabTek chamber slides were treated with TGFβ1 and/or CILP1 for 48 h before fixing in 4% paraformaldehyde and permeabilizing with 1% Triton X-100. Immunocytochemistry was performed using anti-αSMA monoclonal antibody, TRITC-conjugated anti-mouse secondary antibody and DAPI-containing mounting medium (Vector Laboratories) to stain nuclei. Images were obtained under equivalent optical conditions. αSMA ELISA was performed essentially as described previously^30^. Serum-starved primary adult rat fibroblasts were cultured in 96-well tissue culture plates and treated with TGFβ1 and/or CILP1 for 48 h before fixing in methanol for 30 min. Non-specific protein binding sites were blocked in BSA and αSMA was detected using anti-αSMA monoclonal antibody followed by HRP-conjugated anti-mouse secondary antibody and TMB substrate.

### TGFβ-activity analyses

HEK293 cells were plated on 6-well plates at a density of 200.000 cells/well. At 90% confluency and after a 4 h serum starvation period, the cells were transfected with a TGFβ reporter construct, containing SMAD-binding elements (500 ng/well) cloned into the MLP-pGL3 luciferase vector (CAGA-Luc)^17^ and co-transfected (500 ng/well) with the pCMV-SPORT6-hCilp1 construct (Open Biosystems) using Lipofectamine 2000 (Invitrogen). As a control for transfection efficiency CMV-β-galactosidase pON249 was co-transfected (250 ng/well). 24 h post-transfection the cells were incubated with TGFβ1 for 24 h (1 ng/ml). Luciferase activity as a measure of SMAD-binding activity was measured in the cell lysates using the SteadyGo firefly luciferase assay (Promega) on the Victor plate reader (PerkinElmer). β-Galactosidase activity was determined spectrophotometrically on the Victor plate reader.

### Statistics

Data are presented as mean±SEM and were analysed with Student’s t-test or ANOVA with Bonferroni’s multiple comparison test where appropriate. Correlations were calculated using Pearson’s method. Differences were considered statistically significant when p < 0.05.

## Electronic supplementary material

supplemental info
