# Supplementary material for: Cartilage intermediate layer protein 1 (CILP1): A novel mediator of cardiac extracellular matrix remodelling
Source: Sci Rep. 2017 Nov 22;7:16042. doi: 10.1038/s41598-017-16201-y (PMC5700204; doi:10.1038/s41598-017-16201-y)

Supplemental data

Cartilage intermediate layer protein 1 (CILP1):

A novel mediator of cardiac extracellular matrix remodelling

Frans A. van Nieuwenhoven[1*], Chantal Munts[1], Roel C. op 't Veld[1], Arantxa González[2], Javier Díez[2], Stephane Heymans[3], Blanche Schroen[3] and Marc van Bilsen[1]

Departments of [1]Physiology and [3]Cardiology, Cardiovascular Research Institute Maastricht (CARIM), Maastricht University, Maastricht, the Netherlands.

[2]Program of Cardiovascular Diseases, University of Navarra, Pamplona, Spain.

Supplemental Table 1. Baseline characteristics of study participants

Characteristics	CABG (n=6)	AoS (EF > 55%; n=6)	AoS (EF < 55%; n=5)
Age (y)	66.7 ± 3	68.5 ± 3	71 ± 2
Men/women (n)	4/2	3/3	5/0
LV EDD (mm)	47.2 ± 2	46.2 ± 2	58.8 ± 4* #
LV WT (mm)	8.9 ± 0.5	12.5 ± 1**	9.7 ± 0.4
EF (%)	59.7 ± 2	64.0 ± 2	45.6 ± 3** ###
CILP1 (mean array intensity)	329 ± 109	427 ± 73	518 ± 65

Data are shown as average ± SEM
CABG, Coronary Artery Bypass Graft; AoS, Aortic stenosis; LV EDD, left ventricular end-dyastolic diameter; LV WT, left ventricular wall thickness; EF, ejection fraction
*p <0.05; **p <0.01 vs CABG
#p <0.05; ###p <0.001 vs AoS EF >55%

Supplemental Table 2. Gene-specific primer sequences used for quantitative real-time PCR

Gene	Species	Forward primer	Reverse primer
alpha-Smooth muscle actin (ACTA2)	rat	AAGGCCAACCGGGAGAAAAT	AGTCCAGCACAATACCAGTTGT
Cartilage intermediate-layer protein 1 (CILP1)	mouse	ACAGGGAGCAGAGACCAG	GACCAGGAACTCCAGATGTG
Cartilage intermediate-layer protein 1 (CILP1)	rat	GAGTACTTCTGTAAGGCGCAG	GGCATTCTGGAAGCAATCATG
Cartilage intermediate-layer protein 1 (CILP1)	human	AATTACACCGTACGCTTCCTC	CATCTCTGCCAAGCAAATGC
Collagen, type 1, alpha 1 (Col1A1)	mouse	CGAAGGCAACAGTCGCTTCA	GGTCTTGGTGGTTTTGTATTCGAT
Collagen, type 1, alpha 1 (Col1A1)	rat	CGAAGGCAACAGTCGATTCA	GGTCTTGGTGGTTTTGTATTCGAT
Collagen, type 1, alpha 1 (Col1A1)	human	AGGACAAGAGGCATGTCTGGTT	GGACATCAGGCGCAGGAA
Connective tissue growth factor (CTGF)	mouse / human	CACAGAGTGGAGCGCCTGTTC	GATGCACTTTTTGCCCTTCTTAATG
Cyclophilin-A (Cyclo)	mouse / rat	CAAATGCTGGACCAAACACAA	TTCACCTTCCCAAAGACCACAT
Cyclophilin-A (Cyclo)	human	CCCACCGTGTTCTTCGACAT	CCAGTGCTCAGAGCACGAAA
Transforming growth factor, beta 1 (TGFβ1)	mouse	ATCCTGTCCAAACTAAGGCTCG	ACCTCTTTAGCATAGTAGTCCGC
Transforming growth factor, beta 3 (TGFβ3)	mouse	GGACTTCGGCCACATCAAGAA	TAGGGGACGTGGGTCATCAC

Supplemental Table 3. Cilp1 correlation factors

Number	Array_ID	Entrez_ID	Gene Name	r	r^2
1	1440709	8483	CILP	1.0000	1.0000
2	4040671	1278	COL1A2	0.9096	0.8274
3	2360576	NA	NA	-0.8922	0.7961
4	2600164	7058	THBS2	0.8913	0.7944
5	5860152	960	CD44	0.8911	0.7941
6	6330504	56256	SERTAD4	0.8877	0.7879
7	4180301	22891	ZNF365	0.8821	0.7781
8	4290431	1809	DPYSL3	0.8801	0.7746
9	240400	56937	PMEPA1	0.8794	0.7734
10	4200674	3485	IGFBP2	0.8784	0.7717
11	5290132	80781	COL18A1	0.8741	0.7640
12	7650672	23452	ANGPTL2	0.8699	0.7567
13	4560328	6624	FSCN1	0.8687	0.7547
14	5090626	2191	FAP	0.8665	0.7508
15	4290292	NA	NA	-0.8658	0.7496
16	1430278	1513	CTSK	0.8656	0.7492
17	460767	633	BGN	0.8638	0.7461
18	1030008	7043	TGFB3	0.8637	0.7459
19	2360551	220	ALDH1A3	0.8637	0.7459
20	1110048	11341	SCRG1	0.8630	0.7448
21	5960021	5305	PIP4K2A	0.8620	0.7431
22	4570398	NA	NA	0.8609	0.7411
23	6590341	11031	RAB31	0.8563	0.7332
24	5870424	57616	TSHZ3	0.8559	0.7325
25	1230039	9068	ANGPTL1	0.8548	0.7307
26	6900053	8495	PPFIBP2	0.8534	0.7284
27	940735	4256	MGP	0.8533	0.7281
28	6760593	114884	OSBPL10	0.8527	0.7271
29	7400368	861	RUNX1	0.8506	0.7236
30	3130615	10016	PDCD6	0.8485	0.7200
31	5080192	5270	SERPINE2	0.8469	0.7172
32	2120021	1281	COL3A1	0.8466	0.7168
33	1070114	26508	HEYL	0.8443	0.7129

34	1470669	3371	TNC	0.8428	0.7103
35	2470114	1293	COL6A3	0.8401	0.7058
36	4810575	9719	ADAMTSL2	0.8380	0.7022
37	630601	4735	SEPT2	0.8363	0.6994
38	6290356	5176	SERPINF1	0.8356	0.6982
39	2470079	7756	ZNF207	0.8327	0.6934
40	3940435	2012	EMP1	0.8295	0.6881
41	4900520	7857	SCG2	0.8258	0.6820
42	3370162	10418	SPON1	0.8255	0.6814
43	1770685	4628	MYH10	0.8237	0.6785
44	1400044	259217	HSPA12A	-0.8190	0.6708
45	5720180	2535	FZD2	0.8190	0.6707
46	6770309	4637	MYL6	0.8189	0.6706
47	4220239	NA	NA	0.8179	0.6690
48	6980669	219438	OR5D18	-0.8170	0.6674
49	6330008	NA	NA	0.8166	0.6669
50	4640743	1266	CNN3	0.8164	0.6665
51	610164	2534	FYN	0.8164	0.6664
52	6580711	165	AEBP1	0.8143	0.6631
53	2100274	54843	SYTL2	0.8131	0.6611
54	830376	NA	NA	0.8121	0.6595
55	4120386	5118	PCOLCE	0.8109	0.6575
56	2760181	51805	COQ3	-0.8101	0.6563
57	6180554	79987	SVEP1	0.8078	0.6526
58	3830762	338773	TMEM119	0.8065	0.6505
59	5720327	5684	PSMA3	-0.8063	0.6502
60	4490292	1290	COL5A2	0.8062	0.6499
61	6220019	5179	PENK	0.8050	0.6481
62	2510091	1295	COL8A1	0.8047	0.6476
63	6420025	55107	ANO1	0.8035	0.6457
64	3440386	1893	ECM1	0.8035	0.6456
65	5720438	493869	GPX8	0.8022	0.6435
66	6840639	255631	COL24A1	0.7966	0.6346
67	5490431	6303	SAT1	0.7950	0.6320
68	4920202	4130	MAP1A	0.7948	0.6317
69	6040750	NA	NA	-0.7938	0.6301

70	1510646	8277	TKTL1	-0.7922	0.6276
71	5570139	25797	QPCT	0.7919	0.6271
72	2510392	79188	TMEM43	0.7913	0.6261
73	2340131	NA	NA	-0.7913	0.6261
74	4830575	151887	CCDC80	0.7910	0.6257
75	5820646	10241	CALCOCO2	-0.7910	0.6257
76	3890138	10903	MTMR11	0.7905	0.6248
77	50128	199857	ALG14	0.7897	0.6236
78	3780438	NA	NA	0.7894	0.6232
79	3190021	59277	NTN4	0.7894	0.6232
80	1980671	55188	RIC8B	-0.7891	0.6227
81	4220440	10788	IQGAP2	0.7889	0.6223
82	2190377	8666	EIF3G	-0.7888	0.6222
83	70167	23643	LY96	0.7883	0.6214
84	2650112	6567	SLC16A2	0.7878	0.6207
85	6420731	6574	SLC20A1	0.7873	0.6199
86	6200253	NA	NA	0.7870	0.6194
87	3610102	2307	FOXS1	0.7864	0.6185
88	2970445	NA	NA	0.7864	0.6184
89	270706	1287	COL4A5	0.7855	0.6171
90	3850131	10971	YWHAQ	0.7852	0.6166
91	4590441	1727	CYB5R3	0.7852	0.6165
92	6980129	221476	PI16	0.7839	0.6145
93	3940440	11214	AKAP13	0.7838	0.6143
94	2970730	91663	MYADM	0.7836	0.6141
95	70196	1296	COL8A2	0.7823	0.6120
96	1470296	3908	LAMA2	0.7806	0.6094
97	7150563	NA	NA	0.7805	0.6091
98	4040544	2042	EPHA3	0.7797	0.6080
99	2140228	1039	CDR2	0.7794	0.6075
100	3780228	7188	TRAF5	0.7790	0.6069
101	4670544	55902	ACSS2	-0.7753	0.6011
102	6620008	3730	ANOS1	0.7749	0.6005
103	5690463	79703	C11orf80	0.7745	0.5999
104	5960725	2887	GRB10	0.7743	0.5995
105	2710114	2782	GNB1	0.7739	0.5989

106	1510458	4627	MYH9	0.7734	0.5981
107	1690487	285382	C3orf70	0.7727	0.5971
108	830278	152007	GLIPR2	0.7719	0.5958
109	450253	5747	PTK2	0.7713	0.5950
110	610452	3688	ITGB1	0.7688	0.5911
111	5260561	7216	TRO	0.7685	0.5907
112	3310424	71	ACTG1	0.7672	0.5886
113	5910113	1462	VCAN	0.7658	0.5864
114	4860546	115908	CTHRC1	0.7658	0.5864
115	1660598	65267	WNK3	0.7653	0.5856
116	6760475	1284	COL4A2	0.7642	0.5840
117	2230161	57142	RTN4	0.7638	0.5834
118	540075	10577	NPC2	0.7632	0.5825
119	6450291	7044	LEFTY2	0.7627	0.5817
120	6650189	5830	PEX5	-0.7626	0.5815
121	2260241	NA	NA	-0.7619	0.5805
122	5420754	NA	NA	0.7618	0.5804
123	5820278	644378	NA	-0.7613	0.5796
124	7380279	79709	COLGALT1	0.7603	0.5781
125	940474	27286	SRPX2	0.7590	0.5761
126	6100482	493	ATP2B4	0.7585	0.5752
127	4760427	23287	AGTPBP1	-0.7560	0.5715
128	4250689	NA	NA	-0.7555	0.5708
129	5290246	9213	XPR1	0.7551	0.5701
130	3140603	6840	SVIL	0.7548	0.5697
131	6760661	726	CAPN5	0.7523	0.5659
132	2140121	10409	BASP1	0.7523	0.5659
133	2140242	7130	TNFAIP6	0.7522	0.5658
134	6980739	25890	ABI3BP	0.7516	0.5649
135	6350367	10647	SCGB1D2	-0.7514	0.5646
136	4010215	337979	KRTAP22-1	0.7511	0.5642
137	7050220	10874	NMU	-0.7511	0.5641
138	2630437	64094	SMOC2	0.7508	0.5637
139	20647	84168	ANTXR1	0.7502	0.5629
140	20288	55752	SEPT11	0.7500	0.5625
141	2190326	1734	DIO2	0.7498	0.5621

142	1090128	NA	NA	0.7495	0.5617
143	150551	NA	NA	0.7493	0.5615
144	6480703	558	AXL	0.7485	0.5603
145	3170072	23020	SNRNP200	0.7482	0.5598
146	1710408	NA	NA	0.7481	0.5596
147	5900286	146198	ZFP90	0.7479	0.5594
148	3830402	NA	NA	0.7474	0.5587
149	1170647	37	ACADVL	-0.7473	0.5585
150	270128	NA	NA	-0.7465	0.5573
151	6550600	4609	MYC	0.7463	0.5570
152	6020719	51715	RAB23	0.7462	0.5569
153	4610390	60	ACTB	0.7461	0.5567
154	4730133	NA	NA	0.7455	0.5557
155	150328	NA	NA	0.7452	0.5553
156	7380180	79865	TREML2	-0.7450	0.5550
157	6650327	1047	CLGN	-0.7447	0.5546
158	7610730	139818	DOCK11	0.7442	0.5538
159	4640053	NA	NA	-0.7438	0.5533
160	2940450	NA	NA	-0.7436	0.5529
161	2320026	NA	NA	0.7434	0.5527
162	1300280	7852	CXCR4	0.7431	0.5522
163	4780615	302	ANXA2	0.7430	0.5521
164	4480152	4969	OGN	0.7430	0.5521
165	770463	27295	PDLIM3	0.7426	0.5515
166	3870136	8815	BANF1	0.7419	0.5504
167	450398	9371	KIF3B	0.7406	0.5484
168	2470730	NA	NA	0.7398	0.5473
169	3440519	400120	SERTM1	-0.7397	0.5471
170	770564	79762	C1orf115	-0.7389	0.5460
171	4210309	50999	TMED5	-0.7388	0.5459
172	830593	7431	VIM	0.7387	0.5456
173	630377	387	RHOA	0.7381	0.5449
174	1470020	9567	GTPBP1	0.7381	0.5447
175	3390647	NA	NA	-0.7380	0.5446
176	5290408	84693	MCEE	-0.7377	0.5442
177	5700681	100131735	NA	-0.7375	0.5440

178	2750372	NA	NA	0.7371	0.5433
179	4220441	5789	PTPRD	0.7366	0.5426
180	4540021	506	ATP5B	-0.7365	0.5424
181	2970600	NA	NA	-0.7363	0.5421
182	3420519	NA	NA	-0.7359	0.5416
183	1850193	81689	ISCA1	-0.7357	0.5412
184	290373	85363	TRIM5	-0.7356	0.5412
185	6400148	140606		0.7356	0.5412
186	5360243	3693	ITGB5	0.7342	0.5391
187	4880537	51474	LIMA1	0.7339	0.5386
188	1690044	11238	CA5B	0.7337	0.5382
189	110307	11259	FILIP1L	0.7333	0.5377
190	1440440	7867	MAPKAPK3	-0.7332	0.5376
191	3460278	83606	GUCD1	-0.7328	0.5370
192	2680767	64951	MRPS24	-0.7328	0.5370
193	1030519	4601	MXI1	-0.7325	0.5365
194	4480593	2200	FBN1	0.7323	0.5363
195	610095	1995	ELAVL3	-0.7322	0.5361
196	620440	NA	NA	-0.7321	0.5360
197	4260593	26088	GGA1	0.7320	0.5359
198	3170504	89781	HPS4	0.7318	0.5355
199	5490593	622	BDH1	-0.7317	0.5354
200	870328	51295	ECSIT	-0.7317	0.5354
201	4900193	84186	ZCCHC7	0.7315	0.5350
202	3290551	8672	EIF4G3	0.7309	0.5342
203	2600431	29015	SLC43A3	0.7303	0.5334
204	2650220	11167	FSTL1	0.7302	0.5332
205	6520066	6845	VAMP7	0.7300	0.5329
206	1570370	84681	HINT2	-0.7300	0.5329
207	5550768	NA	NA	0.7294	0.5320
208	2650753	222223	KIAA1324L	0.7293	0.5318
209	3290398	5250	SLC25A3	-0.7292	0.5317
210	460600	NA	NA	0.7285	0.5307
211	1240243	25923	ATL3	0.7279	0.5298
212	1980632	3301	DNAJA1	0.7278	0.5296
213	830369	92912	UBE2Q2	0.7275	0.5293

214	160348	7278	TUBA3C	-0.7275	0.5292
215	4220767	4092	SMAD7	0.7269	0.5284
216	2640543	6424	SFRP4	0.7267	0.5282
217	1260040	2202	EFEMP1	0.7264	0.5276
218	7380619	3835	KIF22	-0.7259	0.5269
219	650328	7293	TNFRSF4	0.7258	0.5267
220	4220041	6558	SLC12A2	0.7251	0.5258
221	6330593	5745	PTH1R	-0.7250	0.5256
222	5560180	22872	SEC31A	0.7240	0.5242
223	240494	6932	TCF7	-0.7238	0.5238
224	6130682	2875	GPT	-0.7231	0.5229
225	4670059	51776		0.7230	0.5228
226	3800215	9721	GPRIN2	0.7230	0.5227
227	6770403	NA	NA	-0.7229	0.5225
228	7150475	9948	WDR1	0.7228	0.5224
229	4060279	10758	TRAF3IP2	0.7225	0.5220
230	6110195	219654	ZCCHC24	0.7224	0.5219
231	2940075	55437	STRADB	-0.7223	0.5218
232	3190689	64062	RBM26	0.7218	0.5209
233	2030553	NA	NA	0.7216	0.5208
234	1440707	10513	APPBP2	0.7204	0.5190
235	5360743	1307	COL16A1	0.7204	0.5189
236	4220064	2821	GPI	-0.7200	0.5184
237	6960142	1277	COL1A1	0.7196	0.5179
238	4230520	8655	DYNLL1	0.7195	0.5177
239	6350392	6509	SLC1A4	0.7193	0.5174
240	7380450	NA	NA	0.7189	0.5168
241	3400538	3490	IGFBP7	0.7185	0.5162
242	3120520	11345	GABARAPL2	0.7182	0.5158
243	1570075	26272	FBXO4	0.7181	0.5156
244	50300	NA	NA	-0.7180	0.5155
245	6020315	NA	NA	-0.7179	0.5154
246	7510349	NA	NA	0.7173	0.5146
247	4290390	7412	VCAM1	0.7164	0.5132
248	4200259	47	ACLY	0.7158	0.5123
249	4490017	51228	GLTP	0.7156	0.5121

250	3800066	595135	PGM5P2	0.7156	0.5121
251	4220148	NA	NA	-0.7149	0.5111
252	5690687	NA	NA	0.7149	0.5111
253	3190400	56971	CEACAM19	0.7149	0.5111
254	4780050	7358	UGDH	0.7147	0.5107
255	4070152	NA	NA	-0.7145	0.5105
256	1010176	81544	GDPD5	0.7142	0.5100
257	270458	83690	CRISPLD1	0.7141	0.5100
258	6940037	3357	HTR2B	0.7140	0.5098
259	50037	157378	TMEM65	0.7135	0.5091
260	1980360	51150	SDF4	0.7134	0.5089
261	3440132	NA	NA	-0.7133	0.5088
262	5130750	3087	HHEX	0.7132	0.5087
263	650678	6876	TAGLN	0.7128	0.5082
264	290162	NA	NA	0.7121	0.5070
265	6550315	57149	LYRM1	-0.7120	0.5069
266	7050445	NA	NA	0.7119	0.5068
267	6520451	7096	TLR1	0.7116	0.5063
268	5960528	51104	ABHD17B	-0.7116	0.5063
269	580465	22846	VASH1	0.7113	0.5060
270	2190475	26052	DNM3	0.7104	0.5047
271	6770025	2197	FAU	-0.7104	0.5047
272	630327	5979	RET	-0.7098	0.5038
273	2190674	NA	NA	0.7097	0.5037
274	6130577	2006	ELN	0.7095	0.5034
275	3360064	255189	PLA2G4F	-0.7093	0.5032
276	7550398	NA	NA	-0.7090	0.5027
277	4150603	NA	NA	0.7076	0.5007
278	650332	NA	NA	-0.7076	0.5007
279	2600519	85395	FAM207A	-0.7076	0.5006
280	5340504	9465	AKAP7	-0.7075	0.5006
281	2360066	253935	ANGPTL5	0.7072	0.5001

NA indicates not annotated

Gene names according to HGNC

Supplemental Table 4. Enrichment analysis of genes of which the expression correlated strongly (r2>0.5) with CILP1 using the PANTHER Overrepresentation Test (release 20160321). GO Ontology database released 2016-04-23.

Number	GO number	Biological Proces	Fold Enrichment	P-value
1	50849	negative regulation of calcium-mediated signaling	27.21	1.19E-02
2	30574	collagen catabolic process	16.26	3.72E-09
3	30199	collagen fibril organization	16.04	3.12E-03
4	44243	multicellular organism catabolic process	14.87	1.21E-08
5	32963	collagen metabolic process	14.34	1.94E-08
6	44259	multicellular organismal macromolecule metabolic process	13.55	4.10E-08
7	31032	actomyosin structure organization	12.9	2.41E-06
8	44236	multicellular organism metabolic process	12.56	2.02E-08
9	30239	myofibril assembly	11.95	2.16E-02
10	22617	extracellular matrix disassembly	11.33	1.71E-08
11	71230	cellular response to amino acid stimulus	11.29	3.12E-02
12	71300	cellular response to retinoic acid	10.72	9.24E-03
13	6635	fatty acid beta-oxidation	10.51	4.95E-02
14	48013	ephrin receptor signaling pathway	8.34	1.53E-02
15	30198	extracellular matrix organization	8.15	2.28E-17
16	32526	response to retinoic acid	8.14	5.06E-03
17	43062	extracellular structure organization	8.13	2.48E-17
18	71559	response to transforming growth factor beta	7.49	6.77E-06
19	71560	cellular response to transforming growth factor beta stimulus	7.18	4.07E-05
20	71229	cellular response to acid chemical	7.18	4.07E-05
21	1649	osteoblast differentiation	6.97	2.00E-02
22	1503	ossification	6.57	1.51E-06
23	10632	regulation of epithelial cell migration	6.33	5.30E-03
24	43123	positive regulation of I-kappaB kinase/NF-kappaB signaling	6.32	1.83E-03
25	7179	transforming growth factor beta receptor signaling pathway	6.31	4.74E-02

Supplemental figure S1: Relative CILP1 expression levels in patients receiving coronary artery bypass graft (CABG, n=6), or with aortic stenosis (AoS, subdivided in patients with ejection fraction, EF> 55% (n=6) and patients with EF < 55%(n=5)). Individual data points with means are presented.

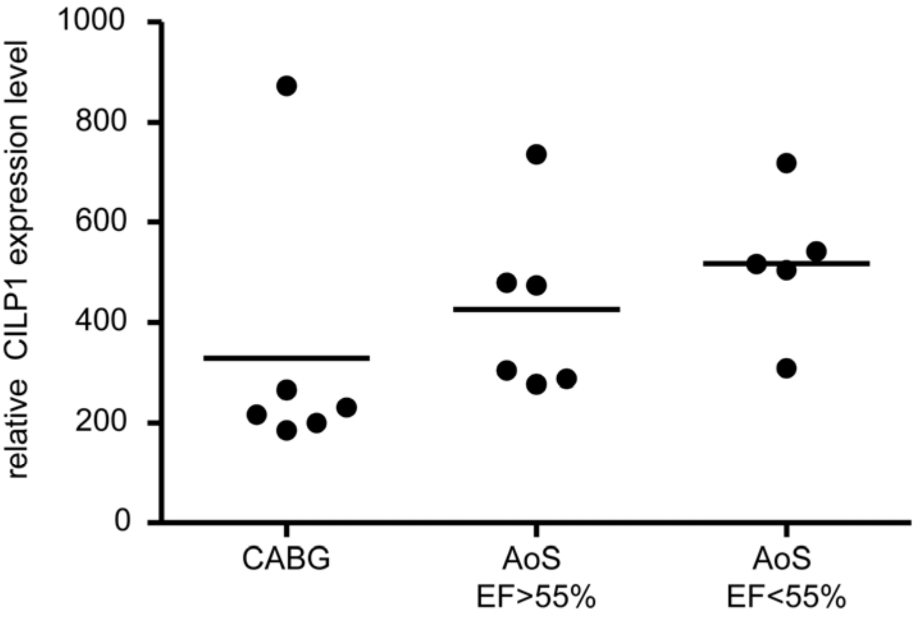

Supplemental figure S2: Relative cardiac left ventricular expression level of CILP1, CTGF, Col1a1, TGFβ1 and TGFβ3 in control animals (C, n=9) and in animals after 4 weeks of AngII infusion (n=9). Data are expressed as mean ± SEM. * $p<0.05$; *** $p<0.001$

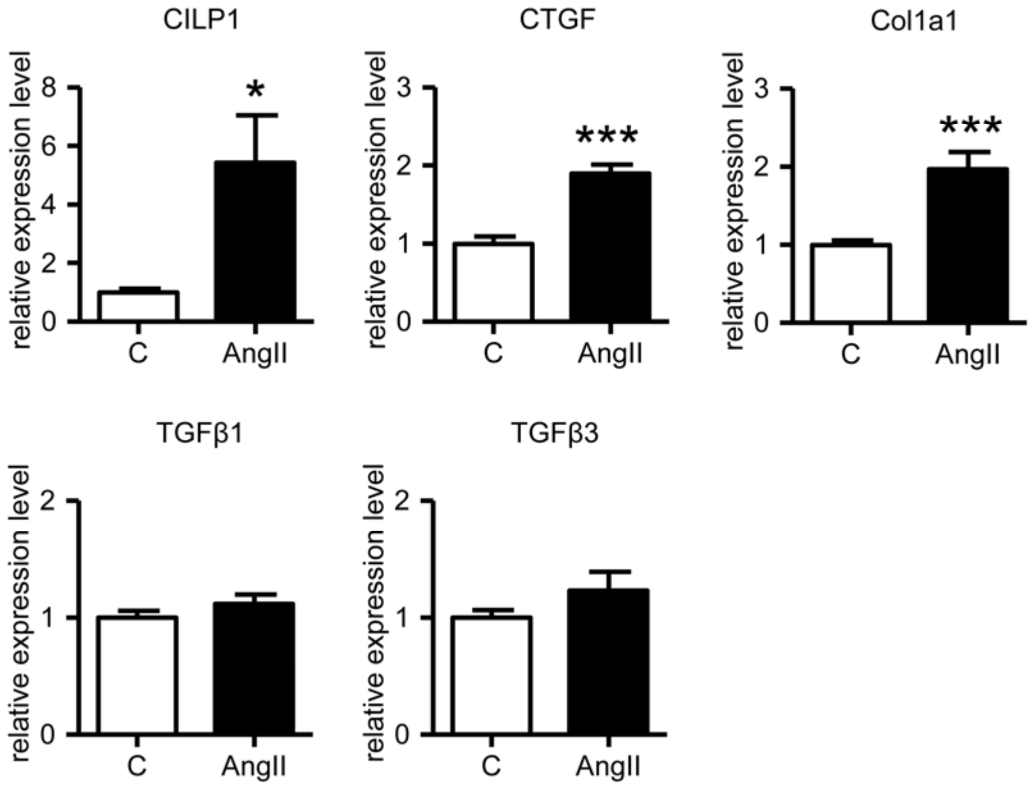

Supplemental figure S3: Relative cardiac left ventricular expression level of CILP1, CTGF, Col1a1 and TGFβ1 in control animals (C, n=5) and in animals at 4 weeks after transverse aortic constriction (TAC, n=6). Data are expressed as mean ± SEM. * $p<0.05$

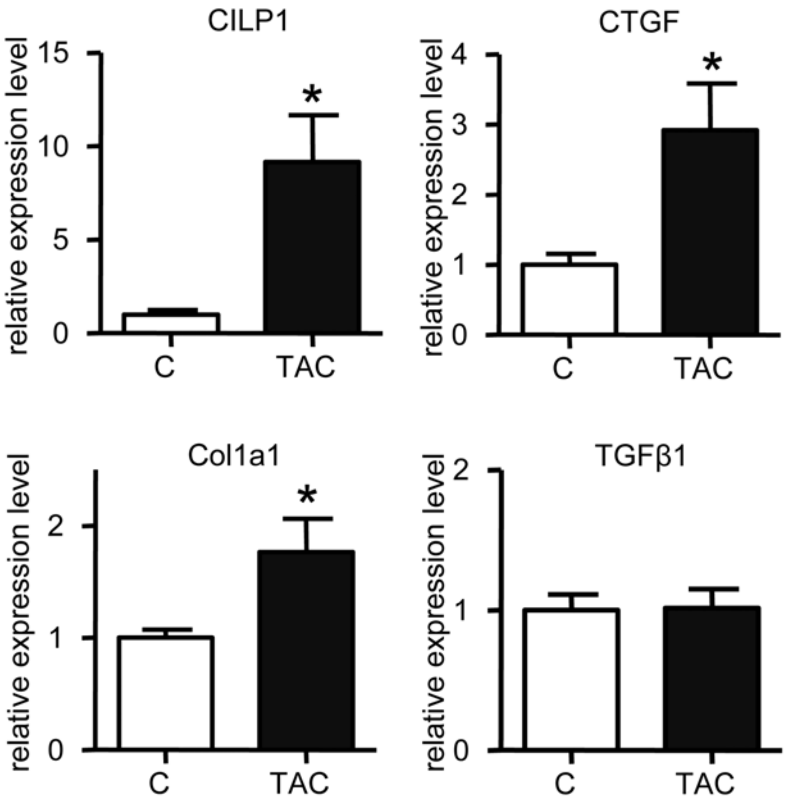

Supplemental figure S4: Effect of TGFβ1, IL-1 and IGF-1 incubation for 24 h on gene expression levels of CILP1 and ACTA2 in primary adult rat ventricular fibroblasts. Data are from 5 separate isolations and are expressed as mean ± SEM. * p<0.05; ***p<0.001

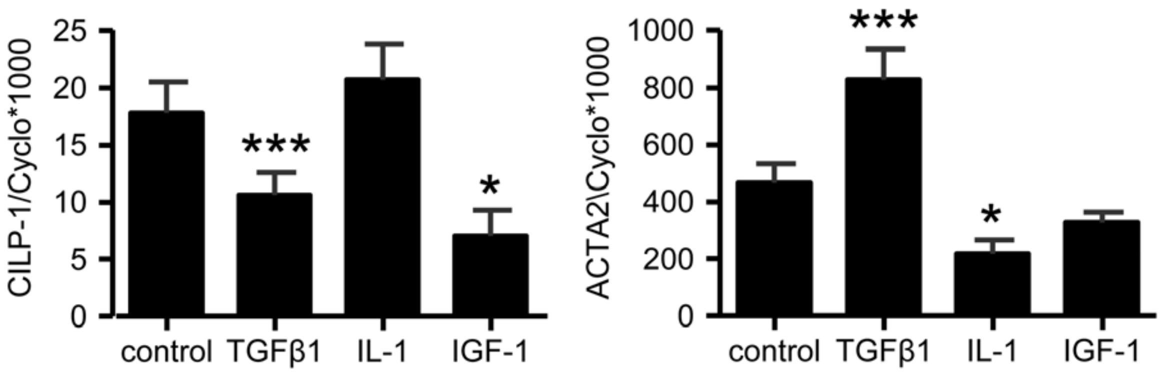

Supplemental figure S5: Detection of CILP protein in conditioned media from transfected HEK293 cells by Western blot analysis.
Lane 1, recombinant human CILP1 (10 ng)
Lane 2, human cartilage 3 µg
Lane 3, Conditioned medium from HEK293 cells transfected with CILP expression vector
Lane 4, Concentrated conditioned medium from HEK293 cells transfected with CILP expression vector.

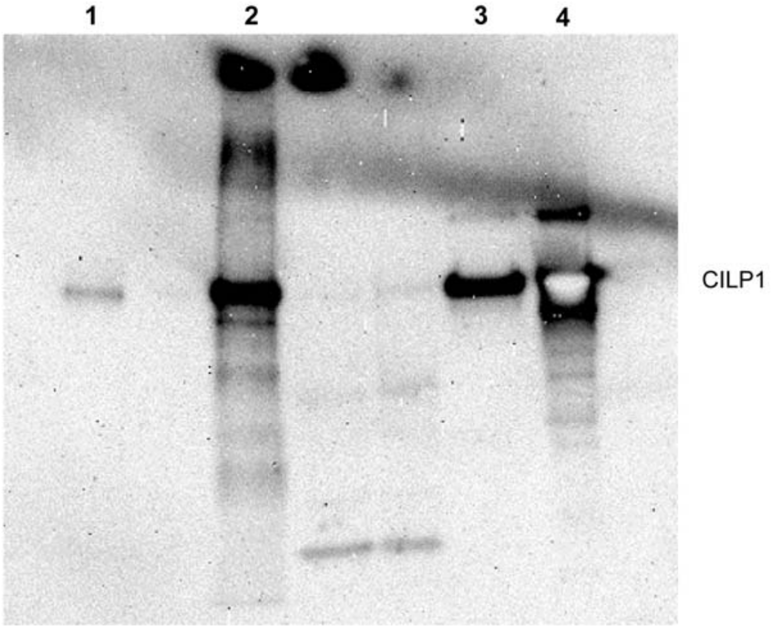

Supplemental figure S6: Full-length western blot shown in Figure 1A CILP expression in human myocardial infarct tissue. CILP1 protein was detected in human myocardial tissue by Western blot analysis.
Lane 1, human cartilage 5 µg
Lane 2, human cartilage 2.5 µg
Lane 3, empty
Lane 4, human cardiac left ventricular tissue
Lane 5, human infarct tissue
Lane 6, human infarct tissue
Lane 7, human infarct tissue
Lane 8, human infarct tissue

Note that the tissue samples from lane 4 and 5 were obtained from the same human subject.

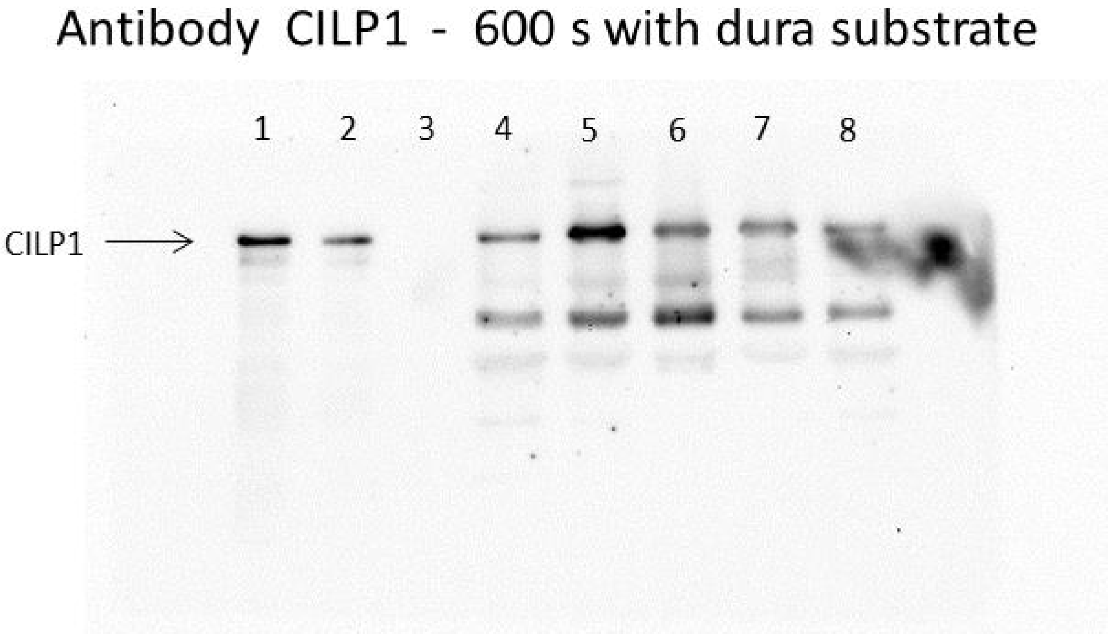

Supplemental figure S7: Full-length western blot shown in Figure 1B
Western blot of endomyocardial biopsies from patients with aortic valve stenosis and control subjects.
Lane 1, Control subject spiked with 1 ng recombinant CILP1
Lanes 2-7 Aortic valve stenosis (AS) patients
Lanes 8-10, control subjects.

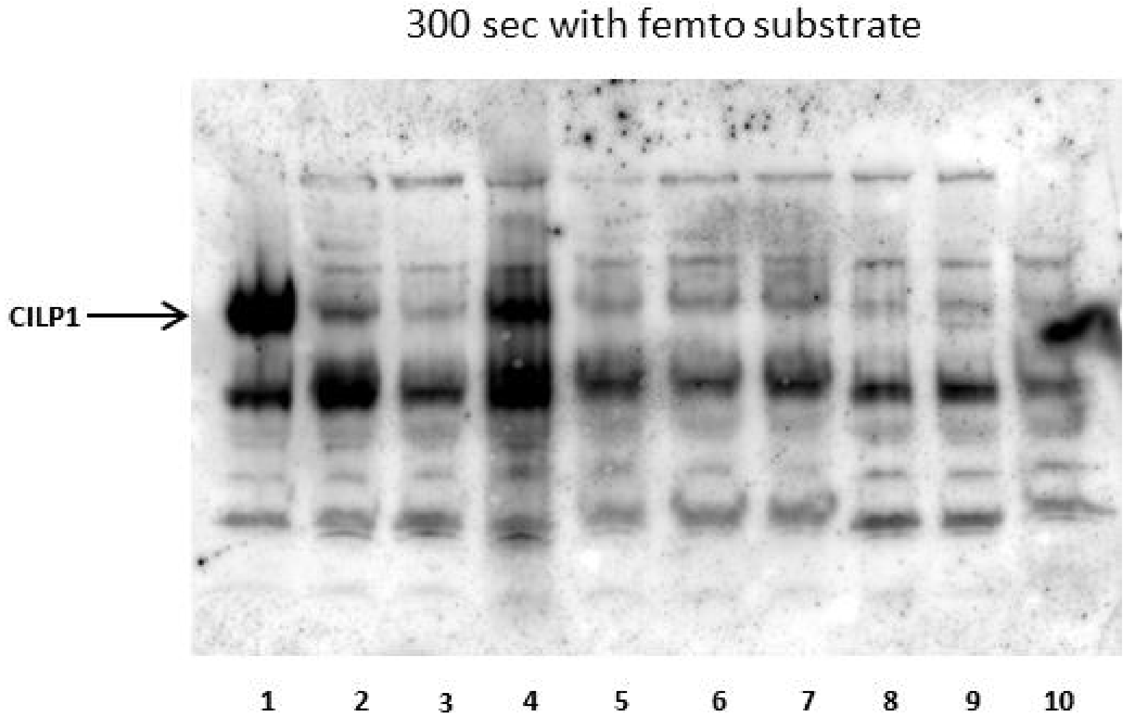

Supplement: Supplementary file 1 — supplemental info [file 41598_2017_16201_MOESM1_ESM.pdf]
